# Effect of Biomass Torrefaction on the Syngas Quality Produced by Chemical Looping Gasification at 20 kW_th_ Scale

**DOI:** 10.1021/acs.energyfuels.4c01096

**Published:** 2024-06-13

**Authors:** Oscar Condori, Alberto Abad, María T. Izquierdo, Luis F. de Diego, Ibai Funcia, Raúl Pérez-Vega, Juan Adánez, Francisco García-Labiano

**Affiliations:** † Instituto de Carboquímica (ICB-CSIC). Miguel Luesma Castán 4, 50018 Zaragoza, Spain; ‡ National Renewable Energy Centre, Av. Ciudad de la Innovación 7, 31621 Sarriguren, Spain

## Abstract

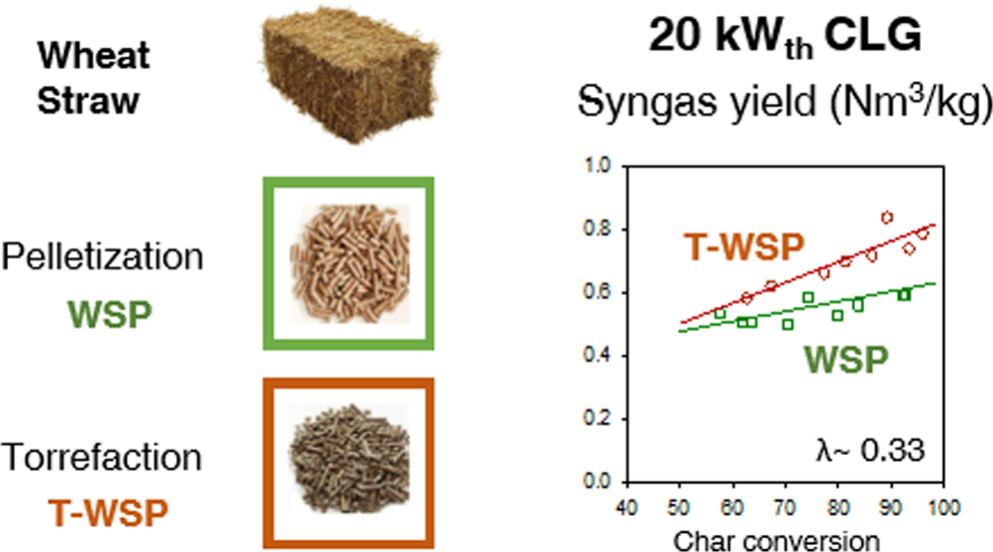

The innovative Biomass Chemical Looping Gasification (BCLG) process uses two reactors (fuel and air reactors) to generate nitrogen-free syngas with low tar content under autothermal conditions. A solid oxygen carrier supplies the oxygen for partial oxidation of the fuel. This study investigated the BCLG process, conducted over 25 h of continuous operation at 20 kW_th_ scale, using ilmenite as the oxygen carrier and wheat straw pellets as fuel (WSP). The effect of using torrefied wheat straw pellets (T-WSP) on the syngas quality was assessed. In addition, the impact of several operational variables on the overall process performance and syngas yield was analyzed. The primary factors influencing the syngas yield were the char conversion through gasification and the oxygen-to-fuel ratio. Higher temperatures, extended residence times of solids in the fuel reactor, and using a secondary gasifier led to increased char conversion, enhancing H_2_ and CO production. Optimizing the air reactor design could enhance the CO_2_ capture potential by inhibiting the combustion of bypassed char. While char conversion and syngas yield with T-WSP were lower than those with WSP at temperatures below 900 °C, T-WSP achieved a higher syngas yield under conditions favoring high char conversion. The presence of CH_4_ and light hydrocarbons showed minimal sensitivity to operating conditions variation, limiting the theoretical syngas yield. Overall, the CLG unit operated smoothly without any agglomeration issues.

## 1 Introduction

According to the latest Intergovernmental Panel on Climate Change (IPCC) report, net greenhouse gas (GHG) emissions have increased constantly over the past decade in all of the relevant sectors. The main causes were related to the increase of fossil fuel use as a primary energy source due to economic and population growth, urbanization, and increased mobility. Approximately, in 2019, 33% of the total emissions were attributed to the energy sector, 24% to industry, 22% to agriculture, forestry, and other land uses, 15% to transport, and 6% to buildings.^1^ The transport sector represents a significant contributor to global GHG emissions into the atmosphere, where road vehicles account for 70% of total direct emissions of this sector, while aviation, shipping, and rail represent 12, 11, and 1%, respectively.^1^ To address this issue and mitigate part of the total transport emissions, the use of biofuels coming from renewable energy sources has been identified as an option to replace fossil fuels in sectors with difficulties to replace the use of liquid fuels, such as aviation. This would contribute to the goal of achieving a 6% share of energy as sustainable aviation fuel (SAF) by 2030, as outlined by the European Union in the Renewable Energy Directive (RED II) of 2018,^2^ and for keeping the global temperature increase below 2 °C (preferably 1.5 °C) targeted by the Paris Agreement.^3^

To achieve these goals, one potential pathway is through the synthesis of advanced biofuels from biomass-based residues.^4^ Synthesis of biofuels from biomass gasification—a widely recognized thermochemical conversion technology—is a relevant option to produce a wide variety of liquid fuels with tunable characteristics depending on its use.^5^ Gasification is an endothermic process where the energy demand is provided by the partial combustion of the fuel.^6^ Conventionally, either air or oxygen is used for this oxidation. However, in the case of using air, the quality of the resulting syngas is reduced due to dilution with nitrogen. On the other hand, when using oxygen, the incorporation of an air separation unit (ASU) is necessary, thereby increasing the overall cost of the process in both scenarios.

One of the explored alternatives for biomass gasification, up to capacities of 100 MW, is the dual fluidized bed (DFB) technology that employs two interconnected fluidized bed reactors: the combustion and gasification reactors,^7^ enabling the production of high-quality syngas under autothermal conditions without requiring an ASU. The DFB process relies on generating the heat required for gasification by the combustion of part of the fuel char in the combustor and using an inert solid as bed material to transfer sensible heat from the combustion reactor to the gasification reactor. Nonetheless, it is important to note that, although a higher-quality syngas is achieved under autothermal conditions, the exhaust gas from the combustor emits CO_2_ into the atmosphere as a consequence of burning part of the fuel, specifically char, to generate the heat necessary for fuel gasification. Another alternative also based on two fluidized bed reactors is the chemical looping gasification (CLG) process. Unlike the DFB process, CLG uses a solid oxygen carrier as a bed material instead of an inert one in order to avoid CO_2_ emissions at the combustion reactor, being one of the main advantages of the process. As will be discussed later, in the case of the CLG, the heat required for fuel gasification is generated by the oxidation of the oxygen carrier in an air atmosphere instead of burning part of the char and therefore avoiding CO_2_ emissions into the atmosphere.^8^

**Figure 1:**
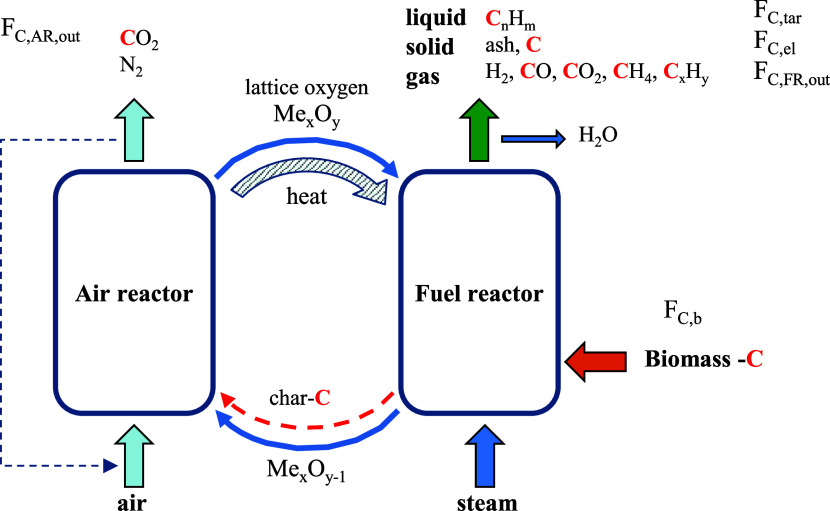
Biomass chemical looping gasification (BCLG) process diagram.

The biomass chemical looping gasification (BCLG) process is a promising technology that could contribute to the decarbonization of the transport sector by producing syngas for the production of a wide range of biofuels, such as gasoline, diesel, methanol, ethanol, naphtha, etc., from biomass with efficient CO_2_ capture.^5,9,10^ This process also relies on a pair of interconnected fluidized bed reactors: the air reactor (AR) and the fuel reactor (FR). In contrast to the DFB process, it employs a solid oxygen carrier, mainly composed of metal oxides (Me_*x*_O_*y*_), as the circulating bed material between the reactors. This oxygen carrier performs two functions by transporting lattice oxygen and the necessary heat from the air reactor to the fuel reactor, facilitating the endothermic reactions occurring in the fuel reactor. This design effectively prevents the mixing of gases between the two reactors.^11^ As shown in Figure 1, the BCLG process enables the efficient conversion of biomass into high-quality syngas with suitable properties to be further processed into synthetic fuels.

The BCLG process offers several advantages over conventional gasification methods, namely:

(a) Undiluted syngas is produced without the need for an air separation unit (ASU). This means that the syngas produced in the BCLG process is nitrogen-free, which increases the heating value of the produced gas.^8^
(b) The BCLG process has been shown to improve the syngas quality by producing a gas with low tar content.^12^
(c) The syngas yield is improved compared to conventional gasification processes, mainly related to the higher conversion of tar compounds. This means that more syngas, which is a mixture of carbon monoxide (CO) and hydrogen (H_2_), can be produced from the same amount of biomass feedstock.^13^
(d) Ideally, no carbon is present in gases from the AR, i.e., gases that will be emitted into the atmosphere. Thus, BCLG helps in confining a fraction of the C-fuel as CO_2_ in gases from the FR. By separating this CO_2_, which may be a step integrated in the synthesis of the liquid fuel, e.g., the Fischer–Tropsch process, this process follows the bioenergy with carbon capture and storage concept (BECCS), and it could potentially result in net-negative emissions.^9^

The chemical processes involved in BCLG maybe briefly described by reactions R1–R7. First, the biomass fuel is introduced into the FR, where it undergoes drying and pyrolysis/devolatilization, resulting in volatile gases (mainly hydrogen, carbon monoxide, carbon dioxide, steam, hydrocarbons, methane, light hydrocarbons, and tars) and solid char; see reaction R1. Subsequently, a series of concurrent reactions take place, involving the gasifying agent, volatilization products of the biomass, and oxygen carrier. The carbon contained in the char is gasified by either H_2_O or CO_2_, which may be used as the fluidizing gas; reactions R2 and R3. Gaseous products may undergo partial oxidation by the oxygen carrier, while the oxygen carrier undergoes reduction concurrently following reactions R4–R6. Other reactions, not described here, may also happen in the FR modifying the gas composition, namely, reforming, cracking, or water–gas shift reaction.^14^

The whole process in the FR is endothermic. The reduced oxygen carrier circulates to the AR where it is regenerated through exothermic oxidation reactions with air; see reaction R7, to start a new cycle. Consequently, lattice oxygen and the heat required for gasification reactions are transported from the AR to the FR by the circulating oxygen carrier, as shown in the process diagram in Figure 1.
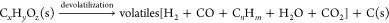
R1
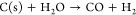
R2
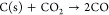
R3
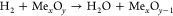
R4
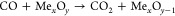
R5
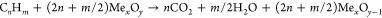
R6
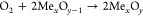
R7This innovative approach allowed the precise adjustment of the oxygen-to-biomass ratio, and it opens the door to the possible widespread adoption of the BCLG technology for syngas production, with high potential to be subsequently utilized in the synthesis of SAF via the Fischer–Tropsch process.^15^ However, the main characteristics of this novel process should be carefully investigated before its scale-up. Several studies have been conducted in continuous CLG units with the objective of evaluating the effect of the operating conditions on the CLG performance, primarily marked by fuel conversion and syngas yield. Some of these variables are temperature, oxygen-to-biomass ratio, and gasifying agent used. Relevant works are compiled in Table 1, where it can be seen the variety of oxygen carriers and fuels used. In most studies, pine sawdust has been used as a biomass fuel because it is an abundant and available resource for large-scale biomass gasification applications. Pine forest residue, wheat straw, almond shell, olive stone, and rice husk are another type of biomass resource that have been studied for BCLG processes. Most of these tests have been conducted with low-cost materials as the oxygen carrier and with the FR being a bubbling fluidized bed. However, similar results can be achieved when the FR was a circulating fluidized bed.^26^

**Table 1:** Experimental Studies Carried Out in Continuous CLG Plants

scale (kW_th_)	oxygen carrier	fuel feedstock	oxygen control method	temperature (°C)	FR fluidization regime	tars (g/kg)	ref
1.5	ilmenite	pine sawdust	OCM-1	820–940	bubbling		(14)
	Fe ore	pine sawdust	OCM-1	820–940	bubbling		(16)
	Mn ore	pine sawdust	OCM-1	820–930	bubbling		(16)
	LD Slag	pine sawdust	OCM-1	820–930	bubbling		(17)
		olive stone			bubbling		
		almond shell			bubbling		
	20% Fe_2_O_3_/Al_2_O_3_	pine sawdust	OCM-1	820–940	bubbling	0.9–3	(18)
	10% Fe_2_O_3_/Al_2_O_3_	pine sawdust	OCM-1	820–940	bubbling		(19)
	25% Fe_2_O_3_/Al_2_O_3_	pine sawdust	OCM-1	820–940	bubbling	1.2–4.5	(19)
	14% CuO/Al_2_O_3_	pine sawdust	OCM-1	820–930	bubbling	0.35–3.85	(20)
5	hematite	coal	OCM-2	865–915	bubbling		(21)
10	70% Fe_2_O_3_/Al_2_O_3_	pine sawdust	OCM-2	670–900	bubbling		(22)
	70% Fe_2_O_3_/Al_2_O_3_	pine sawdust	OCM-2	750–900	bubbling		(23)
	Fe–Ni/Al_2_O_3_	pine sawdust	OCM-2	760–910	bubbling		(24)
20	ilmenite	wheat straw	OCM-1	800–990	circulating		(25)
	ilmenite	pine forest residue	OCM-1	800–950	circulating		(26)
25	hematite (SiO_2_ diluted)	rice husk	OCM-2	800–900	bubbling		(27)
	NiO-CaO/Al_2_O_3_ (SiO_2_ diluted)	rice husk	OCM-2	650–850	bubbling		(28)
1000	Ilmenite	industrial wood	OCM-1	700–880	circulating		(29)
		pine forest residue					

The tests performed in the CLG unit can be classified by the method of controlling the oxygen-to-fuel ratio, which eventually determines the oxygen supplied in the FR:^30^

(a) OCM-1: oxygen control method based on supplying the required oxygen by controlling the air flow into the AR. Here, the solids circulation rate is set at high values.
(b) OCM-2: oxygen control method based on controlling the oxygen carrier circulation rate. Here, an excess of air is fed to the AR.

It has been determined that the OCM-1 is more precise and feasible to be up-scaled.^31^ In addition to the oxygen-to-fuel ratio, it was observed that the variables with the greatest influence on syngas yield were char conversion, which was dependent on the temperature, and the mean residence time of solids in the FR. This residence time may be varied by modifying either the solid circulation rate or the solid inventory in the FR. Thus, in general, increasing the gasification temperature improved biomass conversion and syngas yield. However, syngas yield was constrained by the presence of CH_4_ in the product gas, consistently remaining below 10% and minimally affected by operating conditions.^25^ For example, Condori et al.^26^ determined that the CH_4_ content remained stable at around 10% regardless of the temperature or the oxygen-to-fuel ratio used. The use of Ni- or Cu-based oxygen carriers reduced its concentration below 5%, thanks to their catalytic reforming properties.^20,28^ Despite Ni-based materials typically showing superior physicochemical characteristics, they are dismissed due to their high cost and high toxicity. After all, the Cu-based material and the ilmenite showed the best performance as a bed material, including a longer lifetime. The low cost and favorable performance of ilmenite prompted further investigation on a larger scale in BCLG processes. Thus, recent studies conducted at a 1 MW_th_ scale successfully demonstrated autothermal operation using the oxygen control method OCM-1, including the recirculation of flue gases from the air reactor.^29,31^

The use of pelletized biomass has some advantages regarding the transport, safety, and handling of the fuel. In a previous work at the 20 kW_th_ scale, it has been determined that the size of the pellets is relevant for the biomass conversion due to the slow gasification of the pellets, attributed to diffusional limitations within the pellets.^26^ Char conversion and syngas yield improved when a smaller particle size was used. Also, it was determined that the design of the AR may have a relevant effect on the CO_2_ capture of the CLG process. Thus, unconverted char in the FR was more prone to burning in an oversized AR, causing a decrease in the CO_2_ capture of the process.^26^ Wheat straw pellets were used in a previous work as fuel feedstock using an oversized AR, and only a few tests under steady-state condition were achieved due to the large volume of solids.^25^ Then, the AR of the 20 kW_th_ unit was modified in order to reduce its volume, to achieve more easily steady-state conditions, which was tested with pine forest residue as a fuel.^26^ Relevant differences in the CLG performance were observed between the use of pine forest residue and wheat straw pellets as the fuel, mainly related to the tar composition and the CO_2_ emitted to the atmosphere.^26^

An aspect that has not been previously investigated is the effect of the pretreatment of the raw biomass through drying or torrefaction processes to enhance the performance of the BCLG process and increase the syngas yield. Generally speaking, the torrefaction pretreatment reduces the volatile matter content in the biomass, which may bring some benefits for the syngas quality obtained in the BCLG process in addition to limit the agglomeration phenomena.^32^ Thus, it is believed that the tar content in syngas could be reduced when torrefied biomass was used, which would benefit the cleaning step required for the fuel synthesis process.

The primary objective of this study was to expand our understanding of the influence of biomass fuel properties on the CLG performance in order to find suitable fuels to produce high-quality syngas. Thus, the effect of biomass pretreatment through torrefaction on process efficiency was investigated, being a novel contribution to the development of this technology. To achieve this, wheat straw in the form of pellets was used as a fuel feedstock, which was fed either in its raw state (WSP) or torrefied form (T-WSP). Ilmenite was used as a low-cost oxygen carrier in a 20 kW_th_ scale BCLG unit. All tests presented in this work are newly executed, including those with WSP, using the modified BCLG unit with a lower AR volume. The impact of various operating variables was explored, such as gasification temperature in the fuel reactor (FR), mean residence time of solids in the FR, and the oxygen-to-fuel ratio, including the influence of using a carbon stripper (CS) between both reactors as a secondary gasifier.

## 2 Experimental Section

### 2.1 Oxygen Carrier: Ilmenite

The ilmenite is a natural ore mainly composed of iron titanium oxide, which is usually found in its reduced form, FeTiO_3_. The mineral was supplied by the company Titania AS (Norway) with a purity of 94.3% as sand-type material. It was selected as oxygen carrier for chemical looping processes due to its good physical-chemical properties such as its mechanical resistance, low tendency to agglomeration, low environmental impact, and low cost.^33^ Fresh ilmenite was sieved to obtain a particle size of +100 to 300 μm, which was used during the experimental campaign. In addition, the fresh particles were calcined at 950 °C in air for 24 h prior to the experimentation to improve their initial properties.^34^

The characterization of the ilmenite was carried out using different techniques, as shown in Table 2. XRD analysis, carried out using a Bruker D8 Advance crystalline powder X-ray diffractometer, showed that the main phases present in the calcined ilmenite were pseudobrookite (Fe_2_TiO_5_), hematite (Fe_2_O_3_) and rutile (TiO_2_), indicating that it reached its highest oxidation state during calcination. The composition was determined by complementing the XRD analysis and the thermogravimetric analysis (TGA): 54.7 wt % Fe_2_TiO_5_, 11.2 wt % Fe_2_O_3_, 28.6 wt % TiO_2_, and 5.5 wt % of other inert compounds. The density was determined by helium pycnometry with Micromeritics ACCUPYC II equipment. The crushing strength of the solid particles was evaluated with a Shimpo FGN-5X dynamometer, averaging the results of 20 tests (measured in Newtons). The porosimetry was measured by mercury porosimetry using the Micromeritics AUTOPORE V equipment following the guidelines outlined in ISO 15901 (1–2–3). The determination of specific surface area was carried out through nitrogen physisorption at 77 K using the Micromeritics ASAP 2020 equipment, applying the BET method to the obtained isotherm, in accordance with the ISO 9277 Standard.

**Table 2:** Physical Properties of Calcined Ilmenite

XRD composition	Fe_2_TiO_5_, Fe_2_O_3_, TiO_2_
particle diameter (μm)	100–300
skeletal density (kg/m^3^)	4100
crushing strength (N)	2.2
porosity (%)	1.2
BET surface (m^2^/g)	0.8
oxygen transport capacity, *R*_OC_ (%)	4.0

The oxygen transport capacity (*R*_OC_) is an important characteristic for the oxygen carrier as it represents the mass fraction of the oxygen carrier used for oxygen transfer and depends on the composition of the material and the crystalline phases participating during the redox reactions. It was determined by TGA using a gas mixture of 5 vol % H_2_ and 40 vol % H_2_O, representing the reducing conditions during BCLG operation.
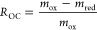
1where *m*_ox_ is the mass of oxidized ilmenite and *m*_red_ is the mass of reduced ilmenite. The calcined ilmenite showed an experimental *R*_OC_ = 4.0 wt % corresponding to the theoretical oxygen transferred by the redox pairs Fe_2_TiO_5_/FeTiO_3_ and Fe_2_O_3_/Fe_3_O_4_, considering the composition of crystalline phases in the oxygen carrier.

### 2.2 Biomass Fuel

During the experimental campaign, two different types of biomass were used as solid fuel. Both biomasses were based on the same feedstock, wheat straw with additives, supplied by CENER (Sarriguren, Spain). Additives were added to increase the ash melting point. The difference was that one of the types of biomass was previously subjected to a thermochemical treatment in the absence of oxygen, defined as torrefaction, to achieve beneficial changes in the biomass composition, such as the reduction of moisture and volatile matter content and the increase of the calorific value. Torrefaction of biomass was carried out at an average temperature of 240 °C for 70 min (including the heating, drying, and torrefaction stages) at the Biorefinery and Bioenergy Centre (BIO2C) of CENER, specifically in the Torrefaction Continuous Pilot Plant. This unit consists of an indirectly heated cylindrical horizontal reactor using thermal fluid as the heating medium, with the combustible gases from the torrefaction reaction burned in a thermal oxidizer. Further description of the torrefaction pilot plant has already been developed elsewhere.^35^ The torrefied wheat straw exited the unit at a rate of around 250 kg/h and with a torrefaction grade, defined by anhydrous weight loss (AWL) parameter, of around 18 wt %. Thus, from now on, they will be defined as wheat straw pellets (WSP) and torrefied wheat straw pellets (T-WSP). The two biomasses were received as pellets (*D* = 6 mm and *L* = 17 mm), and they were fed into the BCLG unit by means of two-screw feeders allowing continuous feeding. The composition differences were obvious in the proximate and ultimate analysis results including the lower heating value (LHV), as shown in Table 3. Besides, the oxygen demand of the fuel (Ω_f_) is another important parameter for the experimental performance, since it represents the stoichiometric moles of oxygen required for biomass combustion as defined by eq 2.
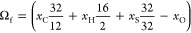
2where *x*_i_ is the fraction of component i in the biomass.

**Table 3:** Analyses of the Fuels Used

	WSP	T-WSP	WSP	T-WSP
proximate analysis (wt %)	as received		dry ash free	
moisture (ISO 18134:2016)	10.3	5.9		
ash (ISO 18122:2023)	6.2	6.7		
volatile matter (ISO 18123:2016)	69.8	67.5	83.6	77.2
fixed carbon	13.7	19.9	16.4	22.8
ultimate analysis (wt %)^a^				
C	41.4	47.6	49.6	54.5
H^b^	5.2	5.6	6.2	6.4
N	0.4	0.6	0.5	0.7
S	0.1	0.1	0.1	0.1
O (by difference)	36.4	33.5	43.6	38.3
low heating value, LHV (kJ/kg)^c^	17,200	17,510	20,350	21,584
Ω_f_ (kg O/kg biomass)	1.16	1.40	1.39	1.60

a Determined in a LECO 628 Series equipment (ISO 16948:2015).

b H does not include hydrogen in H_2_O.

c Determined in a Parr 6400 isoperibolic calorimeter (ISO 18125:2018).

### 2.3 Chemical Looping Gasification Unit

The experimental development of this work was carried out in a chemical looping unit located at the Instituto de Carboquímica (ICB-CSIC). This unit was designed and sized for 20 kW_th_ chemical looping combustion (CLC) or 50 kW_th_ chemical looping with oxygen uncoupling (CLOU) of powdered solid fuels, such as coal.^36^ The versatility of this unit allowed it to be also used for chemical looping gasification (CLG) processes.^25,26^

**Figure 2:**
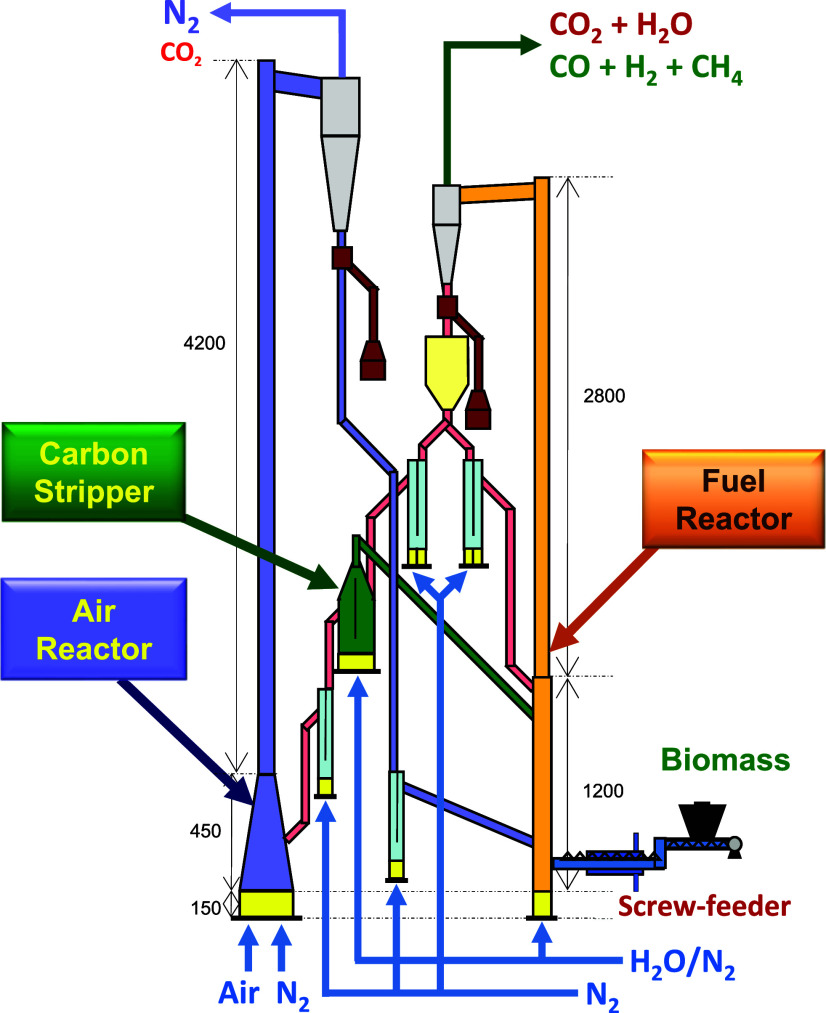
Scheme of the chemical looping unit at ICB-CSIC, used for BCLG at the 20 kW_th_ scale.

As shown in the diagram of Figure 2, this unit consists of two circulating fluidized bed reactors: an air reactor (AR) and a fuel reactor (FR). Both reactors are interconnected by means of loop seals whose main objective is to avoid the gas mixing between both reactors, while allowing the solids circulation. The unit also features a double loop-seal system allowing for an independent oxygen carrier circulation in the FR by recirculating a part of the solids leaving this reactor. However, it was not applied in this work obtaining a global solids circulation rate controlled by the gas flow fed to the AR.

Further, the unit includes a carbon stripper (CS) responsible for separating the unconverted char particles, leaving the FR from the solid oxygen carrier particles. Thus, introducing unconverted char in the FR into the AR would be prevented, avoiding the corresponding CO_2_ emissions from the AR as char would be burned by air. This device was designed for powdered fuels but could not be used to separate char particles with pelletized fuels. In this case, the use of the CS as a secondary gasifier was demonstrated in a previous work with pine forest residue.^26^

The BCLG unit also includes cyclones located at the outlet of each reactor, allowing for the separation of exhaust gases from the circulating solids. It is worth mentioning that this unit incorporates a dedicated system to measure the solid circulation rate.

A CLG campaign with WSP was previously carried out where it was found that an oversize design of the AR caused the use of both high gas flow and high oxygen carrier inventory into the unit to achieve suitable solids circulation.^25^ This resulted in long transition periods to reach a steady state after any changes in any operating conditions. To improve the operability of the system, the AR was modified by decreasing approximately half of the volume by removing the lower cylindrical part of the reactor and expanding the upper conical part, as shown in Figure 2. Then, in this work, new campaigns with WSP and T-WSP were performed.

Straw pellets were fed using a two-screw-feeder system. One of them regulates the biomass feeding rate, and the other is responsible for introducing the biomass as quickly as possible into the fluidized bed to avoid the pyrolysis inside the screw and the consequent clogging.

Nitrogen was used as a fluidizing gas for the different loop seals, while steam was used into the FR, which also acts as a gasifying agent for the biomass. The fluidization of the CS was done with either nitrogen or steam. Nitrogen was used to mimic the performance of an industrial CLG unit without CS. Steam was used to evaluate the performance of CS as a secondary gasified. The fluidization of the AR was carried out with air diluted in nitrogen. Thus, the oxygen transferred to the fuel was controlled by the air flow (OCM-1), while the gas velocity in the AR, required for the solid entrainment, was maintained by adding the corresponding flow of nitrogen.

The composition of key gases exiting both the fuel reactor (CO_2_, CO, H_2_, and CH_4_) and the air reactor (O_2_ and CO_2_) was determined using a continuous analysis system. The concentrations of CO_2_, CO, and CH_4_ are measured using a nondispersive infrared (NDIR) analyzer (Siemens Ultramat 23), while the H_2_ concentration is determined via a thermal conductivity detector (Maihak S710/THERMOR). Besides, the O_2_ concentration is ascertained using a paramagnetic analyzer (Siemens 23/Oxymat 6). In addition, the FR gas line includes a tar collection system, based on the European Tar Protocol.^37^ This setup features a series of 8 impingers filled with isopropanol to absorb moisture and tars, arranged in two separate cooling baths: the first two impingers are kept at 0 °C, while the remaining six are maintained at −20 °C. A cotton filter is placed after the last condenser to capture any tars that might escape from the impingers. A flow meter controls the gas sampling rate provided by a pump, remaining around 1 lN/min, and the total volume of gas passing through the impingers, 60 L, is measured with a gas meter before directing the dry gas to the analyzers. After the experiment is completed, all samples are mixed thoroughly to ensure homogeneity, obtaining a solution with approximately 500 mL of isopropanol containing water and tars. The identification and quantification of tar compounds were performed using a gas chromatograph coupled with a mass spectrometer (Shimadzu GC-2010 Plus + GCMSQP2020). Calibration involved three standards: benzene with 99.8% purity, naphthalene with 99%+ purity, and the EPA 525 PAH MIX-A certified reference material, which contains 13 analytes each at a concentration of 500 μg/mL in dichloromethane. Four different dilutions of the standard reference were prepared to establish the calibration curve. Moreover, the FR gas line includes a sampling system for offline analysis of the light hydrocarbon content (C_1_–C_3_) using a gas chromatograph (PerkinElmer CLARUS 580 GC). Unlike a commercial-scale plant (autothermally heated), this unit performs heating by means of furnaces, allowing the isolation effect of the different operating parameters on the process performance. Pressure and temperature sensors were placed to control the pressure drop and temperature of the reactors and different devices. This makes it easy to determine the distribution of the oxygen carriers within the unit. A detailed description of this unit can be found elsewhere.^26,36^

### 2.4 Procedure and Data Evaluation

One of the main objectives of this work is the study of the effect of different operating variables on the synthesis gas yield and the BCLG process performance represented by different parameters, as shown later. It is expected that the operating variables with the strongest impact on the process efficiency are the temperature, oxygen-to-fuel ratio, and residence time of solids in the FR. Operating conditions are widely shown in Table 4. In general, the temperature was varied in the 850–950 °C interval. Mass balances to the FR and AR were carried out to evaluate the performance of the CLG unit.

**Table 4:** Operating Variables, Performance Parameters, and Gas Composition for the Experimental Campaign at 20 kW_th_ CLG Conditions

	*T*_FR_	λ_FR_	*t*_mr,FR_	Q_CS_	S/B_CS_	*m*_FR_	*m*_FR_^*^	*ṁ*_fOC_	Φ	*X*_f_	*X*_char_	C_AR_	*Y*_sg_	*Y*_sg_^t^	*Y*_HCs_					
test	°C	-	s	L_N_/h	kg/kg	kg	kg/MW	kg/h	-	%	%	%	Nm^3^/kg	Nm^3^/kg	Nm^3^/kg	CO_2_	CO	H_2_	CH_4_	C_2–3_
WSP																				
W-1	932	0.37	149	2000	0.0	6.6	357	160	0.91	87.5	57.6	1.5	0.53	0.90	0.10	41.5	18.7	30.3	9.2	0.2
W-2	948	0.37	277	2000	0.0	8.5	456	110	0.94	99.7	92.3	2.2	0.59	1.08	0.11	40.5	19.9	30.1	8.1	1.3
W-3	900	0.42	257	5000	0.0	5.0	269	70	1.04	87.6	59.8	0.9	0.36	0.81	0.10	50.7	17.1	21.5	8.9	1.8
W-4	920	0.42	259	5000	0.0	5.0	272	70	1.03	94.3	78.7	1.3	0.45	0.93	0.11	46.3	18.9	24.4	8.7	1.6
W-5	934	0.42	207	5000	0.0	5.8	314	101	1.01	92.4	75.4	0.6	0.44	0.91	0.11	46.5	18.8	24.4	8.8	1.5
W-6	939	0.44	145	5000	0.0	6.0	325	150	1.07	89.6	67.3	0.4	0.37	0.83	0.10	50.5	17.3	21.6	9.2	1.5
W-7	947	0.39	179	5000	0.0	4.9	264	98	0.95	94.2	79.7	1.0	0.53	0.99	0.10	41.8	21.0	27.5	8.4	1.3
W-8	854	0.28	230	2000	0.5	9.6	517	150	0.70	88.9	62.0	1.5	0.51	1.07	0.12	41.7	14.3	32.7	8.8	2.5
W-9	857	0.29	258	2000	0.5	9.3	504	130	0.73	92.0	70.6	1.7	0.50	1.11	0.13	42.2	14.6	31.5	8.8	2.9
W-10	858	0.29	218	2000	0.5	9.1	490	150	0.74	90.2	63.8	2.2	0.50	1.06	0.12	41.9	15.1	31.9	8.8	2.4
W-11	894	0.31	374	2000	0.5	8.7	469	84	0.77	95.6	83.7	1.0	0.56	1.14	0.12	41.8	14.7	33.0	8.3	2.3
W-12	933	0.31	177	2000	0.5	7.4	398	150	0.82	95.3	74.3	3.8	0.59	1.09	0.11	39.8	16.9	33.6	8.4	1.4
W-13	936	0.37	321	2000	0.5	7.5	402	84	0.90	98.1	92.5	0.6	0.59	1.09	0.11	42.4	15.5	32.8	7.9	1.3
W-14	944	0.37	213	2000	0.5	8.9	479	150	0.92	98.5	92.1	1.1	0.59	1.08	0.11	42.2	16.5	32.1	8.0	1.2
T-WSP																				
TW-1	934	0.33	949	2000	0.0	5.4	290	18	0.99	94.5	86.3	0.2	0.72	1.20	0.12	37.2	19.0	34.6	7.9	1.2
TW-2	953	0.32	347	2000	0.0	8.9	480	82	0.96	92.9	81.3	0.8	0.70	1.20	0.13	35.8	21.0	33.4	8.5	1.3
TW-3	965	0.35	358	2000	0.0	9.1	494	82	1.04	97.7	93.3	0.6	0.74	1.21	0.12	34.5	24.5	31.6	8.1	1.2
TW-4	889	0.08	1289	5000	0.0	6.1	328	15	0.24	76.3	41.2	0.9	0.89	1.30	0.12	19.8	25.6	44.8	8.5	1.3
TW-5	910	0.20	1482	5000	0.0	7.0	377	15	0.61	86.3	64.9	1.0	0.84	1.25	0.12	25.0	27.6	38.3	8.0	1.2
TW-6	934	0.32	967	5000	0.0	6.1	328	20	0.97	90.9	77.3	0.4	0.66	1.15	0.12	36.4	21.3	32.2	8.8	1.3
TW-7	942	0.31	335	5000	0.0	6.3	341	60	0.92	84.9	62.6	0.6	0.58	0.93	0.12	38.2	19.3	31.6	9.5	1.3
TW-8	945	0.24	197	5000	0.0	10.5	566	170	0.98	65,1	48.6	0.9	0.56	0.92	0.13	34.9	20.7	31.9	10.7	1.8
TW-9	946	0.30	348	5000	0.0	8.7	472	80	0.95	89.6	67.2	3.3	0.62	1.10	0.13	36.2	20.7	32.1	9.7	1.3
TW-10	895	0.32	52	2000	0.5	1.5	80	202	1.08	73.7	35.8	0.5	0.41	0.85	0.11	43.6	17.6	26.9	10.4	1.5
TW-11	899	0.21	271	2000	0.5	6.8	367	80	0.66	87.8	66.2	1.9	0.88	1.28	0.11	30.0	18.9	43.6	6.5	1.0
TW-12	906	0.30	94	2000	0.5	4.0	218	202	1.04	78.6	47.6	0.5	0.57	0.90	0.10	39.0	17.1	34.8	7.6	1.4
TW-13	930	0.35	294	2000	0.5	7.4	398	80	1.03	98.9	96.7	0.3	0.76	1.23	0.13	37.8	17.7	35.8	7.7	1.1
TW-14	935	0.31	189	2000	0.5	3.8	207	64	0.90	96.1	89.1	0.6	0.84	1.25	0.11	33.7	20.3	38.2	6.7	1.1
TW-15	946	0.40	189	2000	0.5	4.8	257	80	1.19	99.3	97.2	0.5	0.71	1.15	0.11	40.1	18.8	33.0	7.0	1.2
TW-16	952	0.34	303	2000	0.5	9.5	514	100	1.00	98.6	95.8	0.3	0.79	1.24	0.12	35.9	20.0	35.6	7.5	1.1

The degree of the oxidation of the fuel is determined by the oxygen transferred in the FR, which is evaluated through the oxygen-to-fuel ratio in the FR, λ_FR_
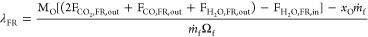
3where F_i_ (mol/s) is the molar flow of the i compound (either at the inlet or outlet of the FR), *ṁ*_f_ (kg/s) is the fuel mass flow fed to the FR, and Ω_f_ (kg O/kg biomass) is the oxygen demand for the full combustion of the fuel feedstock. Also note that *M*_O_ = 0.016 kg of O/mol and *x*_O_ is the mass fraction of oxygen in the fuel.

λ_FR_ is a valuable parameter as it allows the evaluation of the syngas yield by knowing only the conditions in the FR, and it can be done using results both at steady-state and transition periods.^25,26^ However, it was found that after the change in any of the operating conditions, the effective oxygen transference rate in the FR may be different than in the AR during the transition period before reaching the steady state. Thus, the oxygen-to-fuel ratio being transferred in the AR, λ_AR_, was defined by eq 4.
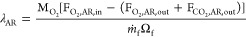
4Further, the relationship between both effective oxygen transfer factors, Φ, allows for evaluating the steady state of the oxygen transfer during the process, assuming values close to 1 (±0.1). These values are shown in Table 4.
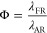
5The mean residence time of solids in the FR, *t*_mr,FR_, directly depends on the global solids circulation rate, *ṁ*_OC_, and the inventory of solids within the FR. The mean residence time of solid was varied by controlling the fluidizing gas velocity into both reactors, which selectively modifies the solids circulation rate and the amount of solids in the FR. Thus, a greater solids entrainment allows for a higher solids circulation rate and consequently a lower residence time.
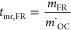
6First, the performance of WSP in the CLG was evaluated; see Table 4. Tests with N_2_ in the CS were carried out in order to evaluate its relevance on char separation without interference of any possible gasification in this reactor. Thus, tests at different temperatures, as well as λ_FR_ and *t*_mr,FR_ values, were performed using two different flows of N_2_ in the CS; see tests W-1 to W-7. Then, tests W-8 to W-14 were carried out by varying the temperature, λ_FR_ and *t*_mr,FR_, but using steam instead of N_2_ to fluidize the CS. Thus, any possible effect of the char gasification in this reactor could be observed.

Second, tests with T-WSP were carried out with the same methodology described for WSP: first, test with two different N_2_ flows in the CS (tests TW-1 to TW-9), and then use of steam in this reactor (tests TW-10 to TW-16).

Regarding the BCLG process performance assessment, the elemental mass balances were initially carried out for each reactor and for the overall process. These balances were based on the results obtained from online and offline gas analysis, and they were used to calculate different parameters describing the BCLG process efficiency. Among the several key performance indicators (KPIs) for the process behavior, it was considered the most representative and most important for this type of processes allowing for the comparison with previous works, as shown in Table 5. These parameters included the following: fuel conversion (*X*_f_), char conversion (*X*_char_), carbon loss to the AR (C_AR_), syngas yield (*Y*_sg_), and hydrocarbons yield (*Y*_HCs_). The values for these parameters are listed in Table 4 for the performed tests. In addition, a theoretical syngas yield (*Y*_sg_^t^) was calculated and is shown in Table 4 considering that CH_4_ and HCs were reformed to H_2_ and CO. More detailed information and discussion about the definition, determination, and use of these parameters can be found in previous studies by the authors.^25,26^

**Table 5:** List of Key Performance Indicators (KPI) Used for BCLG Process Evaluation

parameter	definition
fuel conversion (%)	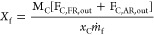 7
char conversion (%)	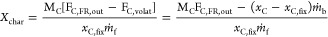 8
carbon loss to the AR (%)	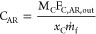 9
syngas yield (Nm^3^/kg)	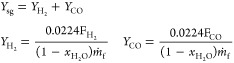 10
HCs yield (Nm^3^/kg)	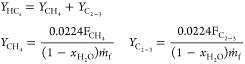 11

## 3 Results

The experimental development of this work was carried out in a BCLG unit at ca. 20 kW_th_ power during 25 h of continuous operation under steady-state conditions using ilmenite as solid oxygen carrier. The time needed to achieve a steady state under specific operating conditions can vary between 15 and 60 min, depending primarily on the preceding conditions, as will be discussed later in Section 3.1. Once the steady state was attained, it was maintained for 30–60 min of stable operation for each test. In general, the CLG unit was smoothly operated, without observing major problems related to defluidization or circulation issues both with WSP and T-WSP fuels. Ilmenite showed good performance with no signs of agglomeration or excessive fines production during continuous operation. A preliminary characterization study showed some migration of iron toward the external surface of the ilmenite particles; however, this did not affect their reactivity, which remained constant during the whole experimental campaign. The possible benefits on the syngas quality by using torrefied wheat straw pellets (T-WSP) will be assessed from the experimental results presented in this work.

The impact of the main operating conditions on the most relevant key performance indicators (KPI) of the BCLG unit is presented in the subsections below. Thus, the variables studied were the temperature in the fuel reactor, mean residence time of solids in the fuel reactor, and oxygen-to-fuel ratio in the FR, λ_FR_. In addition, the behavior of the carbon stripper was evaluated using nitrogen or steam as a gasifying agent, but it is also important to emphasize that in all cases, steam was fed to the FR as a fluidization gas and gasifying agent. Table 4 shows a summary of the results obtained in this work with both types of biomass.

Before entering into the deep analysis of each KPI, a general assessment of the CLG unit performance can be done comparing the global quality of the results previously presented with WSP (using an oversized AR in ref (25)) vs results showed in this work after reducing the AR volume. The modification of the AR was favorable for the smooth operation of the CLG unit, decreasing the duration of the transition periods between different tests by decreasing the global solid inventory in the unit. This allowed us to get a greater number of tests close to the steady state. Thus, the fraction of tests achieving Φ values around the unity increased from 40 to 70%, indicating that the steady state regarding the oxygen being transferred both in FR, λ_FR_, and AR, λ_AR_, was more easily achieved. In addition, the number of tests with a high fuel conversion was also increased. The fraction of tests with fuel conversion values higher than 90% increased from 35 to 80%, and those with *X*_f_ values close to 100% also increased from 16 to 44%. Therefore, a higher number of valuable results could be achieved with a lower experimental effort.

### 3.1 Oxygen Transference Assessment

As mentioned, the BCLG process is based on oxygen transfer from the AR to the FR through a circulating solid oxygen carrier as lattice oxygen. To work under gasification conditions and achieve only partial oxidation of the biomass, this variable must be kept below 1. Samprón et al.^30^ found that the oxygen-to-fuel ratio value should be in the 0.33–0.38 interval in order to achieve the highest syngas yield under autothermal operation. The optimal value mainly depends on the solid circulation rate and the CH_4_ content in syngas. Experimental conditions were varied in order to obtain λ_AR_ and λ_FR_ values at this interval.

**Figure 3:**
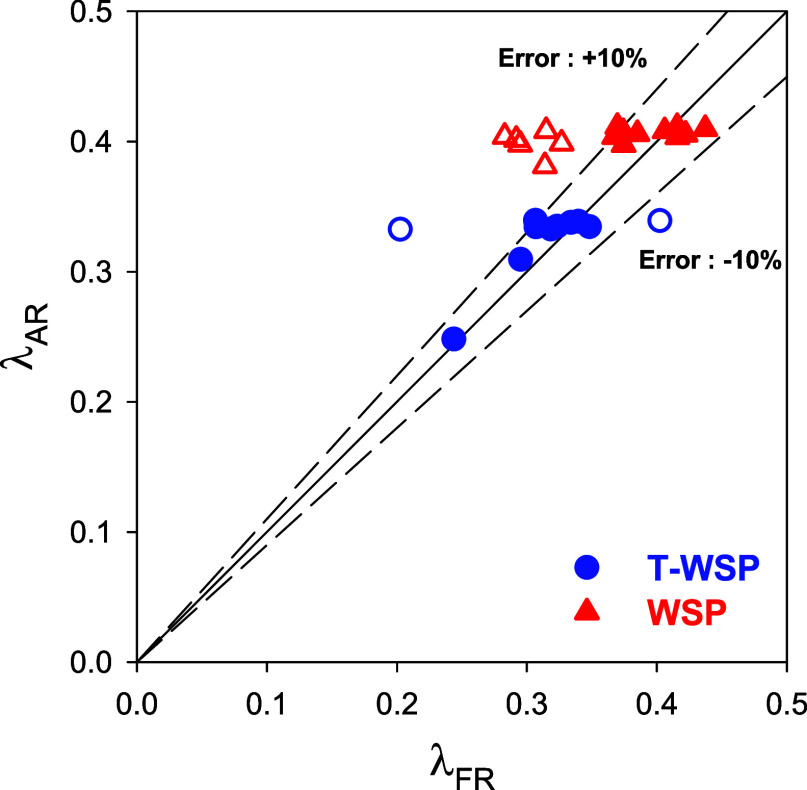
Relationship between the oxygen-to-fuel ratio in AR, λ_AR_, and FR, λ_FR_. Filled dots: steady state conditions: Empty dots: transitory periods.

Figure 3 shows the relationship between the oxygen-to-fuel ratios in the AR and FR, i.e., λ_AR_ and λ_FR_, respectively. When the factor Φ = λ_FR_/λ_AR_ is equal to 1, characterized by the diagonal in Figure 3, the oxygen transfer rate is the same in both reactors, and the steady state has been reached. Considering an error of ±10%, it was observed that most of the tests carried out in this work have reached the steady state with both types of biomass, corresponding to the solid points. On the other hand, the points that are outside this range, empty points, correspond to those tests located in a transition period.

When Φ > 1, the oxygen transfer is higher in the FR than in the AR. This is mainly produced in the transitory period caused by an increase in the gasification temperature in the FR, which increases the gasification rate and the oxygen transfer rate. This variation is more noticeable when prior char accumulation has occurred in the FR under operating conditions that considerably reduce the biomass gasification rate such as low temperatures.

On the other hand, when Φ < 1, higher oxygen transfer is taking place in the AR than in the FR. This phenomenon occurs mainly after a decrease in the FR temperature. In this way, the oxygen carrier is immediately less reduced in the FR, but it still may be oxidized in the AR with the same intensity.

### 3.2 Char Conversion in the Fuel Reactor

**Figure 4:**
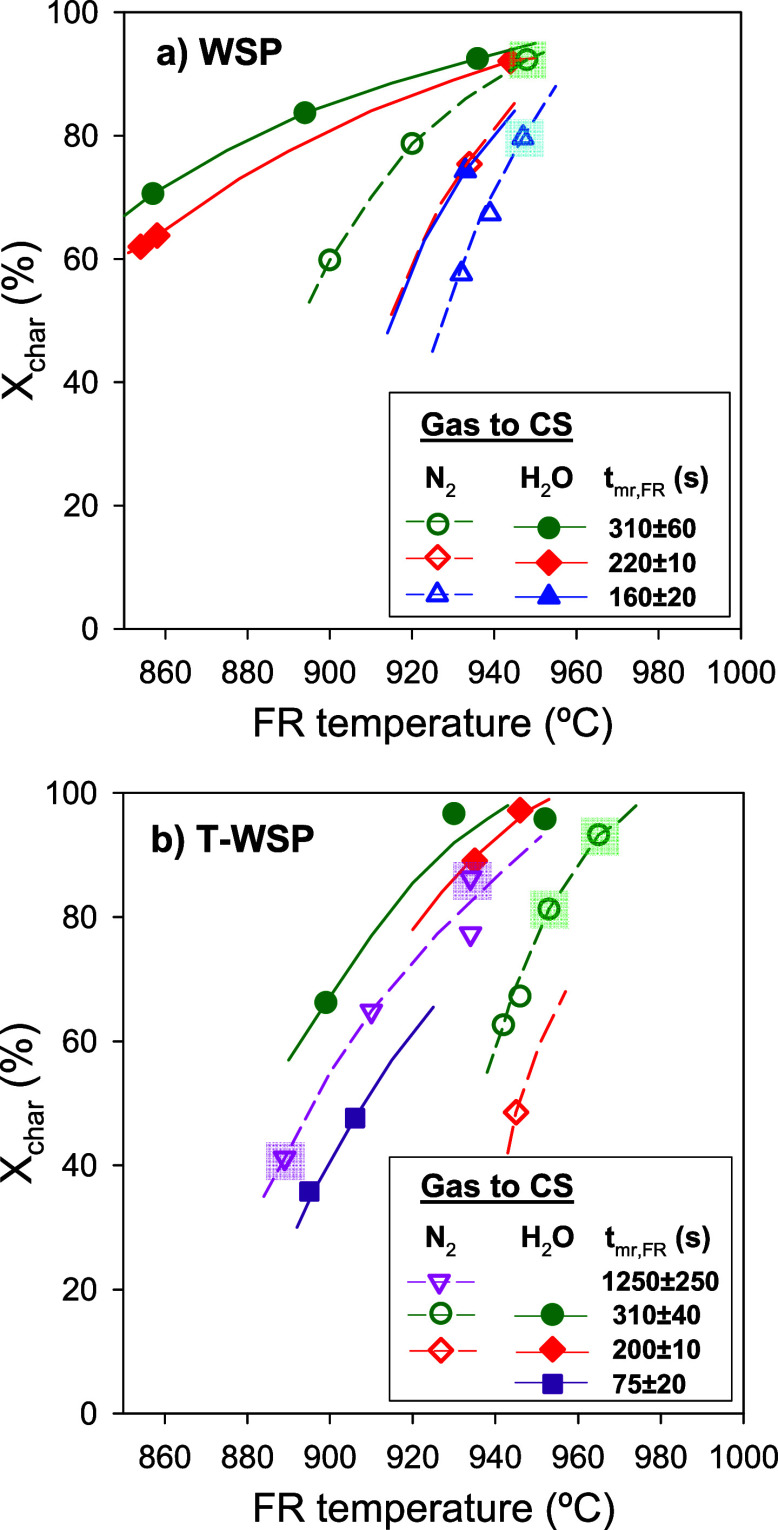
Char conversion, *X*_char_, dependence on the gasification temperature and the mean residence time of solids in the FR using either (a) WSP or (b) T-WSP as fuel feedstock and steam as a gasifying agent in the FR. Q_CS_=5000 LN/h. Highlighted tests corresponds to a Q_CS_=2000 LN/h.

The operating variables with the greatest impact on char conversion were the gasification temperature and the mean residence time of solids in the FR, *t*_mr,FR_. In addition, a series of tests using nitrogen or steam as the fluidizing gas in the CS were performed, while steam was fed into the FR. Figure 4a,b depicts the effect of temperature and *t*_mr,FR_ on char conversion in the FR when feeding nontorrefied (WSP) and torrefied wheat straw pellets (T-WSP), respectively. In the case of using N_2_ in the CS, a quick study on the effect of the gas velocity in the CS was also done in order to corroborate the results previously observed with pelletized biomass.^25,26^ Thus, the purpose of the CS was to separate unconverted char particles from the oxygen carrier, thus hindering the introduction of char in the AR. This unit was designed for powdered fuels. For example, it was demonstrated that an increase of the gas velocity in the CS facilitated the char separation with powdered coal, improving the char conversion in the FR and the CO_2_ capture efficiency.^38^ In the present work, most of the tests were carried out using 5000 LN/h of N_2_ in the CS, corresponding to a gas velocity of 0.35 m/s. In some tests, highlighted in Figure 4, the gas flow was decreased to 2000 LN/h, corresponding to a gas velocity of 0.15 m/s. Considering the general trend of the effect of temperature on the char conversion, there are no indications of a relevant effect of the gas velocity in CS on the char conversion. This result agrees with previous results obtained with pelletized biomass.^25,26^ In this case, the CS was not effective at separating char particles as pellets retained their shape after the partial gasification in the FR; thus, pellets could not be entrained from the CS due to their high terminal velocity. Considering the gas flow into the CS has no relevant effect on the performance of the CLG unit, the rest of the tests were carried out using a gas flow of 2000 LN/h, both with N_2_ and steam.

Figure 4 shows that char conversion increased by either an increase in temperature or an increase in the residence time of solids in the FR with both fuels. An increase in temperature significantly enhances char conversion because it increases the gasification rate. In the case of tests conducted with WSP (Figure 4a) and feeding N_2_ to the CS, the char conversion was higher than 90% in test W-2, characterized by a temperature and residence time of solids in the FR of ca. 950 °C and 280 s, respectively. As N_2_ was fed to the CS, it is expected that the char conversion in this vessel was of low relevance. Then, these results are indicative of what would be expected in a CLG unit without CS. In the case that the CLG unit had a CS, this reactor could be fluidized by steam, which is also a gasifying agent. Thus, in cases where steam was fed to both the FR and the CS, a significant increase in char conversion was achieved, as some of the unconverted char escaping from the FR could be gasified in the CS. For example, compared to test W-2 with N_2_ in the CS, char conversion values higher than 90% could be achieved by operating at a lower temperature (936 °C in W-13) or by having a shorter residence time of solids (213 s in W-14). Therefore, a vessel between the FR and AR can be designed as a secondary gasifier, capable of converting a portion of the char escaping from the FR to recycle the product gases back into the FR, thereby improving the char conversion of the BCLG process.

**Figure 5:**
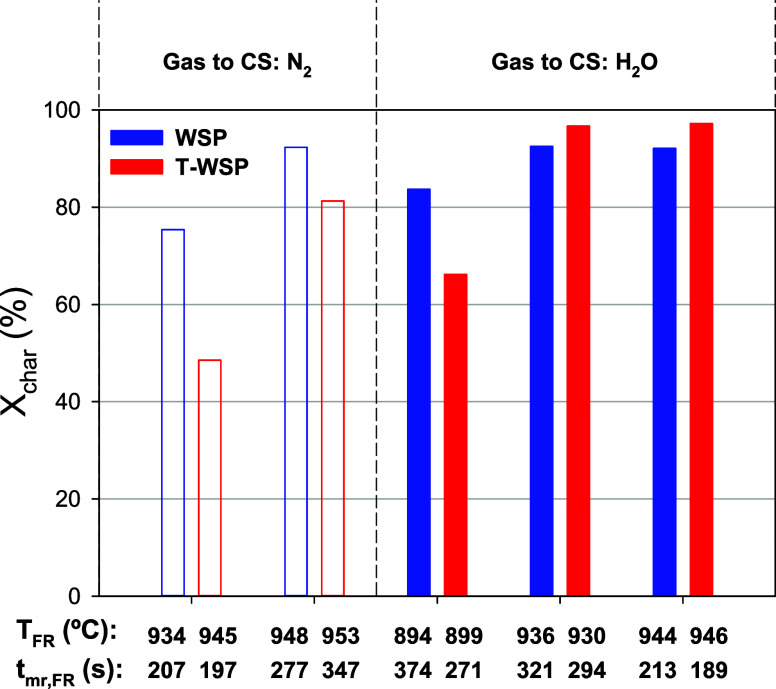
Comparison of the char conversion values achieved with either WSP or T-WSP under different operating conditions.

With T-WSP, Figure 4b, the general trend of the effect of the temperature and mean residence time of solids in the FR on the char conversion was similar to those shown by nontorrefied WSP. However, a closer evaluation of the results highlighted some relevant differences. The slopes of the *X*_char_ vs T_FR_ curves are higher for T-WSP than for WSP, which means that the gasification of the char produced from T-WSP is more sensible to the temperature variation than the char from WSP. In fact, at low temperatures, it is clearly observed that lower *X*_char_ values were obtained with T-WSP. But this difference shortens as the temperature increases. Figure 5 shows a direct comparison of tests performed under similar conditions both with WSP and T-WSP. In general, the char conversion was higher with WSP. But higher *X*_char_ values could be achieved with T-WSP at high temperatures and using steam as fluidizing gas in the CS. This result highlights the relevance of the CS as a secondary gasifier on the char conversion, with it being possible to achieve char conversion values close to 100%.

**Figure 6:**
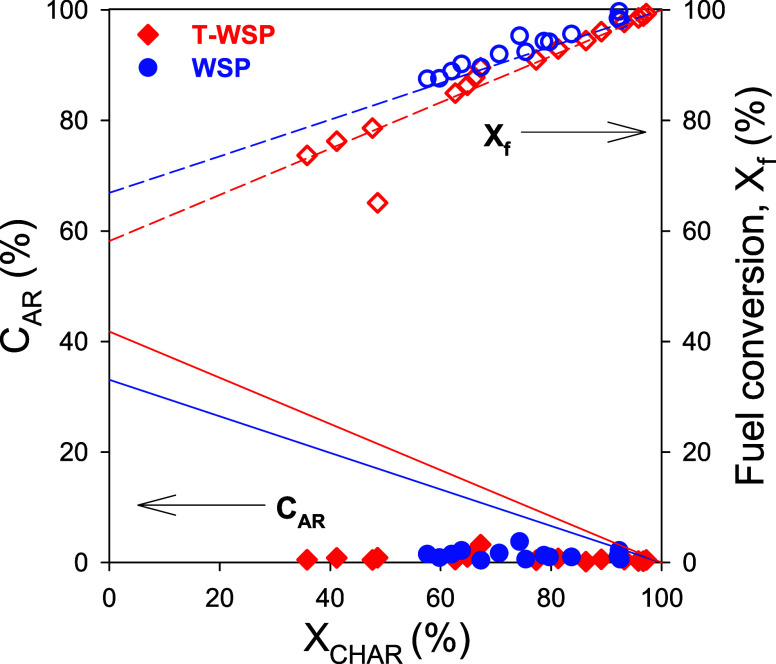
Effect of char conversion in FR and CS, *X*_char_, on the carbon burned in the AR, C_AR_, and the fuel conversion, *X*_f_.

Carbon in unconverted char in the FR and CS may be bypassed to the AR, where it can be burned by air. The fraction of carbon being burned in the AR is emitted as CO_2_, which would affect one of the main advantages of the CLG process related to the carbon compounds confinement at the FR outlet. Figure 6 shows the fraction of carbon in the fuel ending as CO_2_ from the AR. It can be seen that this fraction was low regardless of the char conversion. In theory, C_AR_ would increase as *X*_char_ decreased (indicated by the lines in Figure 6) if all unconverted carbon was burned in the AR. Thus, the oxygen in air fed to the AR has a preference to react with the reduced oxygen carrier instead of burning unconverted carbon.

This fact is a relevant difference compared with results obtained before the AR volume was reduced.^25^ In the previous tests with an oversized AR, the C_AR_ values were usually between 5 and 10%, or even higher values were achieved at low *X*_char_ values. The low C_AR_ values achieved in this work after the reduction of the AR volume suggest that AR should be carefully designed in order to minimize CO_2_ emissions in the CLG process. Thus, char would be recirculated again to the FR with the oxygen carrier particles, which improved the char conversion. This fact was previously observed at the lower 1 kW_th_ scale.^14,16,17^ However, in the 20 kW_th_ unit, the fuel conversion decreased as the char conversion decreased—see Figure 6—which suggests that unconverted char was accumulated somewhere in the CLG unit or escaped from the cyclones. Likely, the AR cyclone was not able to recover the entrained char, which was observed to be shredded to a fine powder after its passage from the AR.

### 3.3 Syngas Yield and Quality

The ultimate desired product in the BCLG process is syngas. Thus, it is essential to convert most of the products from biomass devolatilization into H_2_ and CO. CH_4_, other hydrocarbons, and tar compounds may be also present in the syngas. In this way, the theoretical syngas yield would be experimentally limited by the presence of the HCs in the product gas. The conversion of volatile matter generated from biomass devolatilization, mainly light hydrocarbons or HCs (CH_4_ and C_2_–C_3_), is a key point for increasing the syngas yield. However, these gases often exhibit very low reactivity with most oxygen carriers that lack catalytic capabilities. While the presence of CH_4_ and light hydrocarbons may be easily integrated in the scheme of the synthesis of liquid fuels, e.g., the Fischer–Tropsch process, tars should be previously removed.^15^ Thus, the yields of all of these products should be evaluated.

Typically, in biomass gasification processes, one of the primary objectives is to maximize char conversion, as this indicates a greater production of gases, including H_2_ and CO. As mentioned in Section 3.2, it is important to emphasize the influence of the gasification temperature and the mean residence time of solids in the FR on the char conversion of the process. Thus, it can be said that both variables exhibit an indirect effect on the syngas yield.

**Figure 7:**
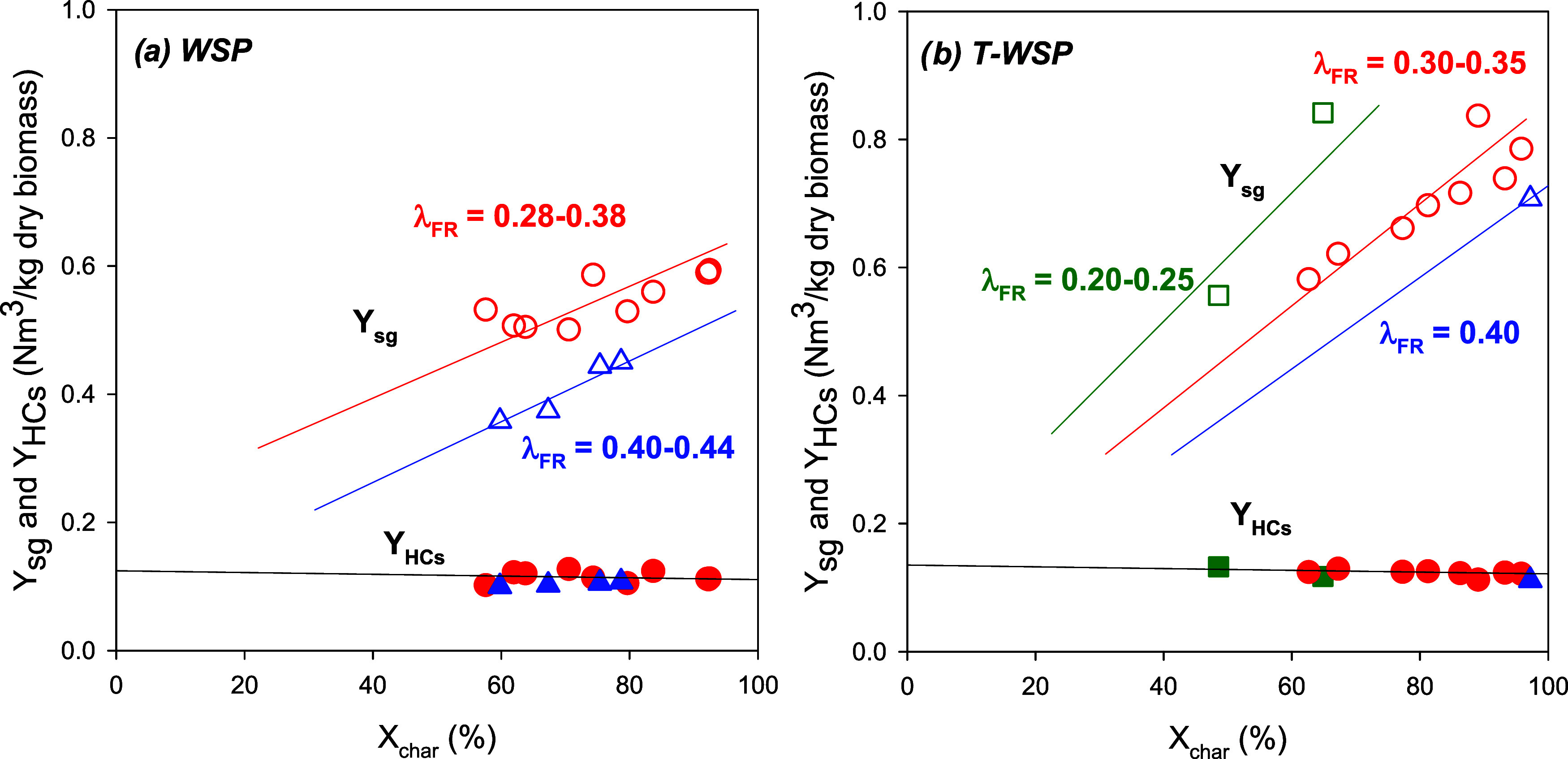
Dependence of syngas yield (*Y*_sg_: empty dots) and hydrocarbons yield (*Y*_HCs_: filled dots) from the oxygen-to-fuel ratio in the FR, λ_FR_, and char conversion, *X*_char_, using both (a) WSP and (b) T-WSP. The proposed trend lines for the green and blue lines in (b) were drawn based on the trend of the red dots. The trends do not go through zero due to the syngas coming from the volatiles.

During the experimentation, the influence of char conversion, *X*_char_, on syngas yield was evident; see Figure 7. Increasing char conversion resulted in a higher syngas yield while maintaining a constant effective oxygen-to-fuel ratio in the FR, λ_FR_. This effect is attributed to the higher production of H_2_ and CO as the primary products of char gasification. Although this trend was observed with both types of biomass, the correlation between *Y*_sg_ and *X*_char_ was more noticeable when torrefied biomass (T-WSP) was used as the fuel feedstock, as depicted in Figure 7b. This difference may be attributed to the compositional variation between the two biomass types, where T-WSP exhibits a higher fixed carbon content and lower volatile matter content. Thus, working under similar conditions, for example, λ_FR_ = 0.3–0.35 and *X*_char_ = 90%, it is observed that it is possible to achieve syngas yields of around 0.8 Nm^3^/kg dry biomass using T-WSP, while the syngas yield with WSP remains around 0.6 Nm^3^/kg dry biomass. Apparently, the pretreatment of biomass through torrefaction can represent an advantage and improve the performance of the BCLG process. The higher density and net calorific value resulting from biomass torrefaction, due to the release of moisture and volatiles, promotes char gasification in the FR, thereby increasing syngas production and reducing CO_2_ emissions in the AR. Additionally, a lower production of light hydrocarbons (C_2_–C_3_) is favored due to the reduced volatile content in the biomass.

On the other hand, analyzing the impact of the λ_FR_ ratio on *Y*_sg_ under similar char conversion conditions, it was observed that the syngas yield decreased with higher λ_FR_ values. This is primarily because a higher oxygen transfer in the FR encourages a greater degree of biomass oxidation or, in simpler terms, the combustion of a larger portion of the syngas generated during char gasification. Similarly, this factor demonstrated a significant influence on the composition of the syngas. Higher λ_FR_ ratios led to decreased concentrations of H_2_ and CO, accompanied by an increase in CO_2_ concentration resulting from the combustion of a larger portion of the syngas.

It is also important to consider the presence of methane and light hydrocarbons (C_2_–C_3_). During experimentation with both types of biomass, it was observed that the presence of HCs in the product gas was hardly affected by variations in the operating conditions. The influence of variables such as λ_FR_ ratio and char conversion—which significantly affect gas composition—on the yield of light hydrocarbons (*Y*_HCs_) was practically negligible, as shown in Figure 7. Considering the difference in volatile content between torrefied and nontorrefied biomass, it is somewhat unexpected not to find significant differences in *Y*_HCs_. In both instances, values of around 0.11–0.12 Nm^3^/kg of dry biomass were achieved. Just as other studies concluded, the main reason points to the low reactivity of ilmenite against compounds such as methane,^33^ which could be also applied to the rest of the light hydrocarbons.

**Figure 8:**
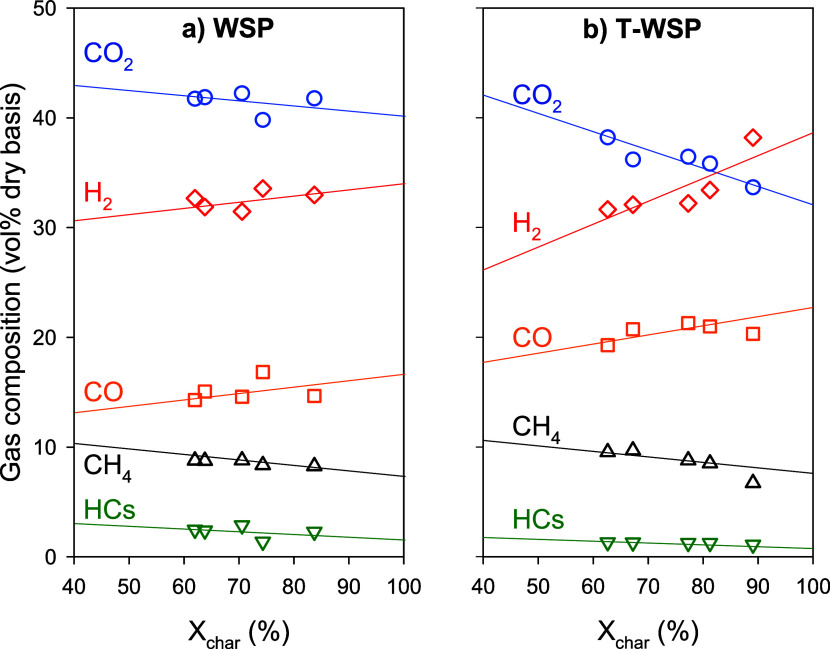
Effect of char conversion on gas composition under similar λ_FR_ ratio conditions for (a) WSP and (b) T-WSP. λ_FR_≈0.30.

Overall, the syngas composition is affected by the operating conditions of the CLG unit; see Table 4. As an example, Figure 8 shows the evolution of the composition of the FR outlet gas as a function of the char conversion for λ_FR_ ≈ 0.3 with both types of biomass. In general, the syngas composition obtained in the BCLG unit did not show significant variations regardless of the biomass type, with CO_2_ being the major compound, followed by H_2_, CO, CH_4_, and other hydrocarbons. However, a closer analysis considering the influence of the char conversion reveals the effect of the different compositions of WSP and T-WSP on the gas composition. Thus, with low char conversions (<50), the gas composition does not show many differences between using one type of biomass or another. But when char conversion is high (>85%), greater differences are observed, with a higher content of H_2_ and CO and a lower content of HCs and CH_4_ with T-WSP.

The main differences between the use of WSP and T-WSP may be described as

(a) Compared to WSP, T-WSP produced a syngas with a slightly higher CH_4_ concentration at the expense of light hydrocarbons.
(b) The syngas produced with T-WSP has a higher amount of CO and lower CO_2_ than that with WSP. The higher carbon content and lower moisture in T-WSP means that more C was introduced for the same mass-based fuel flow, which would result in a higher syngas yield—see Figure 7—and characterized by a higher CO fraction in syngas.
(c) The differences in the fuel composition also affected the variation of the gas composition with the char conversion. The gas composition shows stronger variations with char conversion for T-WSP. Probably, the higher fixed carbon content and lower volatiles in T-WSP make the syngas production more dependent and sensitive to the char conversion achieved during the process. Thus, for T-WSP, the H_2_ content becomes higher than CO_2_ for char conversion values higher than 90%.

**Figure 9:**
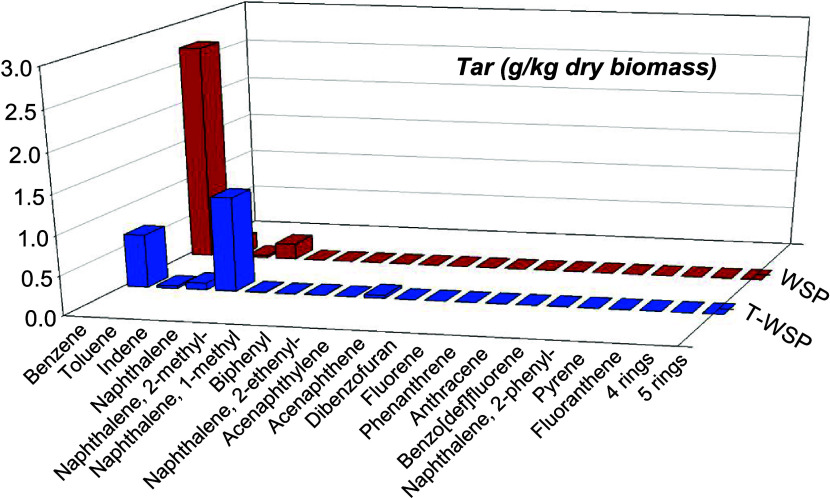
Tar composition distribution for different tests carried out with T-WSP (test TW-16) and WSP (test W-1).

Tar compounds in the produced syngas were also evaluated in selected tests; see Figure 9. Benzene was a compound present in all cases, being the major compound with WSP and with naphthalene, toluene, and indene present in lower concentrations. When T-WSP was utilized, naphthalene became the major compound followed by benzene and smaller traces of indene and toluene.

The total amount of tars generated with WSP was around 3 g/kg of dry biomass, while in the case of using T-WSP, it was around 2 g/kg of dry biomass. This could indicate that another improvement achieved by biomass torrefaction is the reduction of the tar content in the produced syngas.

These results agree with previous works presented in the literature, although they were not carried out in continuous units. In fact, other authors observed that the torrefaction of biomass mainly affects the H_2_/CO ratio, increasing the generation of CO during the gasification process.^39,40^ Fan et al.^40^ found during CLG tests in a fixed bed reactor that the use of torrified eucalyptus wood decreased tar content by one-third, and at the same time increased syngas production.

## 4 Conclusions

The BCLG process was investigated during 25 h of continuous operation at the 20 kW_th_ scale using ilmenite as oxygen carrier and two different types of biomass: wheat straw pellets (WSP) and torrefied wheat straw pellets (T-WSP). High-quality syngas non-nitrogen diluted and with low tar content was obtained during a smooth operation of the CLG unit. The influence of several operating conditions on the process efficiency was evaluated.

A detailed exploration uncovered that the primary factors influencing the syngas yield (*Y*_sg_) were the char conversion through gasification (*X*_char_) and the oxygen-to-fuel ratio in the fuel reactor (λ_FR_). H_2_ and CO production was enhanced by increasing either the FR temperature or the mean residence times of solids in the fuel reactor, *t*_mr,FR_, due to the increase in char conversion, *X*_char_. Both char conversion and syngas yield were improved by using the carbon stripper as a secondary gasifier.

An independent increase in *X*_char_ led to higher syngas yield (H_2_ and CO) while simultaneously increasing the fuel conversion in the CLG unit. Conversely, an independent increase in λ_FR_ resulted in a decrease in *Y*_sg_ by burning part of the syngas and generating a higher CO_2_ content in the product gas. Also, the CO_2_ emissions from the AR were reduced after a reduction of the AR volume because bypassed char to the AR was less prone to burning by air in this reactor. Thus, an optimized design of the AR may enhance the CO_2_ capture potential of the CLG process.

Char conversion and syngas yield with T-WSP was lower than those with WSP at temperatures lower than 900 °C. However, the syngas yield achieved with T-WSP was higher than that with WSP at conditions allowing high char conversion values, e.g., temperature in FR around 950 °C and a mean residence time of solids in the FR of 300 s.

However, the presence of CH_4_ and light hydrocarbons (C2–C3) limited the theoretical syngas yield. The yields of these compounds showed low sensitivity to the variations of the operating conditions, but differences between WSP and T-WSP were found. Thus, the CH_4_ content was higher and C_2_–C_3_ lower for T-WSP. Also, lower tar content was found for T-WSP.

The use of T-WSP as fuel facilitates the achievement of better-quality synthesis gas working under higher *X*_*char*_ conditions compared to WSP. Thus, higher syngas yield was achieved, with the tar content in the syngas being lower from T-WSP.

## Nomenclature

C_AR_: fraction of carbon lost in the air reactor (−)
F_i_: molar flow of i compound (mol/s)
*m*_FR_: solids inventory in the fuel reactor (kg)
*m*_FR_^*^: characteristic solids inventory in the fuel reactor (kg/MW)
*m*_ox_: mass of the oxidized oxygen carrier (kg)
*m*_red_: mass of the reduced oxygen carrier (kg)
M_i_: atomic or molecular weight of i (kg/mol)
*ṁ*_f_: mass flow of fuel fed (kg/s)
*ṁ*_OC_: solids circulation rate between both reactors (kg/s)
Q_CS_: gas flow in the carbon stripper (LN/h)
*R*_OC_: oxygen transport capacity (kg oxygen/kg oxygen carrier)
S/B: steam-to-biomass ratio (kg H_2_O/kg dry biomass)
S/B_CS_: steam-to-biomass ratio in the carbon stripper (kg H_2_O/kg dry biomass)
*t*_mr,FR_: mean residence time of solids in the fuel reactor (s)
T_FR_: temperature in the fuel reactor (°C)
*x*_i_: mass fraction of element i in the biomass (−)
*X*_f_: total carbon conversion of the fuel in the unit (−)
*X*_char_: char conversion (−)
*Y*_i_: yield to i compound (Nm^3^/kg dry biomass)
*Y*_sg_: syngas yield (Nm^3^ syngas/kg dry biomass)
*Y*_sg_^t^: theoretical syngas yield (Nm^3^ syngas/kg dry biomass)
*Y*_HCs_: hydrocarbons yield (Nm^3^ hydrocarbons/kg dry biomass)

## Symbols

λ: overall oxygen-to-biomass ratio (−)
λ_AR_: effective oxygen-to-biomass ratio in the air reactor (−)
λ_FR_: effective oxygen-to-biomass ratio in the fuel reactor (−)
Φ: ratio between the effective oxygen-to-biomass ratio in the fuel and air reactors (−)
Ω_f_: oxygen demand for the full combustion of the fuel (kg oxygen/kg biomass)

## Subscripts

AR: air reactor
FR: fuel reactor
in: inlet stream
out: outlet stream
KPI: key performance indicator
T-WSP: torrefied wheat straw pellets
WSP: wheat straw pellets
